# Advancing the Identification of Risk Factors for Invasive Fungal Disease in Children with Cancer

**DOI:** 10.3390/jof12010060

**Published:** 2026-01-13

**Authors:** Marlon Barraza, Romina Valenzuela, Valentina Gutiérrez, Claudia Greppi, Ana M. Álvarez, Jaime Cerda, María Elena Santolaya

**Affiliations:** 1 Department of Clinical Pharmacy, Hospital Dr. Luis Calvo Mackenna, Santiago 8010037, Chile; mbarraza@calvomackenna.cl; 2 Department of Pediatrics, Centro de Investigación Clínica Avanzada (CICA), Hospital Dr. Luis Calvo Mackenna, Faculty of Medicine, Universidad de Chile, Santiago 8010037, Chile; 3 Department of Pediatric Infectious Diseases and Immunology, Hospital Dr. Sótero del Río, School of Medicine, Pontificia Universidad Católica de Chile, Santiago 8010037, Chile; vgutierrezu@uc.cl; 4 Committee of Infectious Diseases, National Child Program of Antineoplastic Drugs (PINDA), Santiago 8010037, Chile; 5 Department of Pediatrics, Hospital Dr. Roberto del Río, Santiago 8010037, Chile; 6 Department of Pediatrics, Hospital San Juan de Dios, Santiago 8010037, Chile; 7 School of Medicine, Pontificia Universidad Católica de Chile, Santiago 8010037, Chile

**Keywords:** invasive fungal infection, neutropenia, children, cancer, risk factors

## Abstract

Invasive fungal disease (IFD) is one of the leading causes of morbidity and mortality in immunocompromised pediatric patients. This is a multicenter prospective cohort study with a nested retrospective analysis aimed at identifying risk factors for IFD in immunocompromised children with cancer and episodes of persistent high-risk febrile neutropenia (HRFN). One hundred and seventy-four episodes of persistent HRFN were analyzed, of which 34 (19.5%) were confirmed as IFD, 52.9% were caused by filamentous fungi, and 47.1% by yeasts. Logistic regression and survival analyses identified the following significant risk factors for IFD: male sex (OR 4.04), adolescence (OR 4.65), C-reactive protein ≥ 90 mg/L at admission (OR 3.13), and transfer to a critical care unit (OR 10.73). The predictive model demonstrated strong discriminatory capacity (AUC 0.84), with 79.4% sensitivity and 82.1% specificity. These findings highlight that adolescents, particularly males with severe clinical conditions and elevated inflammatory markers, are at the highest risk for IFD during episodes of HRFN. The proposed risk factor-based model may support early risk stratification and guide targeted antifungal prophylaxis or therapy, potentially improving outcomes in this population. Validation an external cohort is required to confirm these results and optimize clinical applicability.

## 1. Introduction

Invasive fungal disease (IFD) is one of the leading causes of morbidity and mortality in both immunocompromised adult and pediatric patients. There are significant differences between these two populations regarding epidemiology, diagnosis, treatment, immune reconstitution capacity following chemotherapy, and comorbidities [1,2]. Therefore, it is not appropriate to use data reported in adults to assess pediatric patients.

One of the most important factors for the survival of immunocompromised patients with IFD is early diagnosis and prompt treatment; in many cases, this is difficult given the non-specific nature of the symptoms and the difficulty of establishing an accurate diagnosis [3]. In this context, treatment is often initiated without certainty that the patient has an IFD, unnecessarily exposing them to antifungal drugs that may be associated with high toxicity, drug–drug interactions, and selection for resistance [2,4].

Children with hematologic malignancies are at increased risk of developing IFD. However, the type of cancer is rarely a single risk factor for the onset of this infection [5]; rather, a combination of factors or interactions between them could increase the risk of developing IFD [6]. Knowledge of these risk factors is essential for the early recognition and treatment of IFD, which is associated with better clinical outcomes [7].

Despite recent advances in the diagnosis and treatment of IFD, morbidity and mortality remain high in pediatric patients with cancer. Hence, different strategies have been developed to address IFD, including empirical therapy, preemptive therapy, and antifungal prophylaxis, each with its own advantages and disadvantages. Empirical antifungal therapy involves the early initiation of antifungal treatment in patients with fever and prolonged neutropenia (>96 h). In contrast, preemptive therapy consists of identifying children at high risk of IFD, formulating an early detection strategy through comprehensive testing, and implementing a more rational and targeted therapy, which is expected to have reduced toxicity while achieving clinical outcomes equivalent to the empirical strategy [4]. This approach has two essential requirements, a highly specialized healthcare team with early clinical suspicion and a thorough and timely diagnostic strategy encompassing clinical, microbiological, molecular, and imaging aspects, which could be a limiting factor in some resource-scarce settings for the application of preemptive approach [8].

Antifungal prophylaxis consists of administering antifungal drugs to populations at high risk of IFD, such as children with acute myeloid leukemia (AML), severe aplastic anemia, leukemia relapses, and allogeneic hematopoietic cell transplant (HCT) recipients during periods of profound and prolonged neutropenia [2,9,10,11]. Prophylaxis is considered a good strategy because it has been shown to reduce the incidence of IFD [11]; however, it remains controversial due to increased risk of opportunistic fungal infections, which are complex and challenging to diagnose and manage [12,13].

An alternative to the aforementioned therapeutic approaches is to develop tools that can predict IFD based on the identification of its risk factors. Some models have been developed to predict IFD in adult patients, but there are few data on pediatric patients [14,15,16,17]. This study aimed to determine the main risk factors for IFD in children with cancer and to evaluate the predictive capacity of these risk factors.

## 2. Methods

### 2.1. Population

We conducted a multicenter prospective cohort study with a nested retrospective analysis. Children under the age of 18 undergoing chemotherapy for cancer who were experiencing episodes of high-risk febrile neutropenia (HRFN) were invited to participate in six hospitals belonging to the National Child Program of Antineoplastic Drugs (PINDA): Dr. Luis Calvo Mackenna, Exequiel González Cortés, Sótero del Río, San Juan de Dios, Roberto del Río, and San Borja Arriarán, 2016–2020, in Santiago, Chile. The study was approved by the Human Research Ethics Committee of the Faculty of Medicine at the Universidad de Chile, and patients were enrolled after their parents signed the informed consent form, and children over the age of 8 gave their assent (FONDECYT 1161662). Patients who developed febrile and neutropenic 96 h after admission were classified as having persistent HRFN and were included in the analyses. According to the Ethics Committee requirements, only one episode of persistent HRFN was selected per patient.

All children with persistent HRFN underwent clinical, microbiological, biomarker, and imaging assessments to diagnosed IFD. All episodes of persistent HRFN were evaluated by an infectious disease expert and classified according to the European Organization for Research and Treatment of Cancer/Mycoses Study Group (EORTC/MSG) definition as proven, probable, possible, and absence of IFD [18]. Episodes of possible IFD and *Pneumocystis jirovecii* infections were excluded from the analyses.

### 2.2. Definitions

(a) Neutropenia: ANC < 500/mm^3^. (b) Fever: single axillary temperature > 38.5 °C or >38.0 °C in two measurements separated by 1 h. (c) HRFN: a febrile neutropenic episode with one or more of the following risk factors at the time of admission: relapse of leukemia as cancer type, hypotension, or quantitative CRP > 90 mg/L, or a febrile neutropenic episode with the following two factors at the time of admission: <7 days between the end of the last chemotherapy and the beginning of the fever and platelet count < 50,000/mm^3^ [4]. (d) Persistent HRFN: fever and neutropenia > 96 h in a child with HRFN. (e) IFD status was defined according to the EORTC/MSG as follows: proven if the child had microbiological or histological evidence of fungal tissue invasion or a positive fungal culture obtained from a sterile site, in addition to clinical or radiological findings consistent with fungal infection; probable, the presence of one or more host factors, a clinical criterion and one or more mycological criterion by a direct test (cytology, direct microscopy or culture) or an indirect test (detection of antigen); and possible, a case meeting host factor and clinical criteria but lacking any mycological documentation. (f) Hypotension: Blood pressure lower than the 5th percentile according to the age or less than 90/50 in children >10 years of age. (g) Sepsis: systemic inflammatory response syndrome in the presence of, or because of, suspected or proven infection, plus one of the following: cardiovascular organ dysfunction, or acute respiratory distress syndrome, or 2 or more other organ dysfunctions: neurologic, hematologic, renal and hepatic. (h) Positive galactomannan: index > 0.5 in serum, >1.0 in bronchoalveolar lavage.

### 2.3. Risk Factors for IFD and Statistical Analysis

For the descriptive analysis, categorical variables were presented as frequencies and percentages, while numerical variables were presented as medians and interquartile ranges (IQRs). Risk factors for IFD were assessed using bivariate and multivariate analyses, including logistic regression and survival analysis.

The association between IFD and the different independent variables was evaluated. The association between categorical variables was assessed using Fisher’s exact test or the chi-square test, as appropriate, along with post hoc analysis (trend test) when necessary. The association with numerical variables was performed using the non-parametric Wilcoxon or Kruskal–Wallis test, as applicable. The crude OR and their respective 95% confidence intervals (CIs) were estimated as a measure of effect using bivariate logistic regression. Event-free survival was evaluated in episodes of persistent HRFN. The follow-up period was defined as the number of days until the patient overcame neutropenia (ANC > 500 cells/mm^3^). Only right-censoring was considered, and total EFS was estimated. Survival was calculated from sociodemographic and admission variables (clinical and laboratory evaluation) using the Kaplan–Meier estimator, and curves were compared using the nonparametric log-rank test. The hazard ratio (HR) and its respective 95% CI were estimated for each variable using the Cox proportional hazard model.

Variables with a *p*-value < 0.1 in the bivariate analyses were used in the multivariate analyses. Logistic regression was performed to estimate the association between IFD and the independent variables (input model). The significant variables in the input model were used to adjust model 1. Adjusted ORs and their respective 95% CIs were estimated. A second model (model 2) was used, adding biological variables of interest as predictors of IFD, as defined by the research group, and was evaluated and compared with model 1.

Given the low prevalence of IFD, the predictive ability of the models was evaluated using the same cohort. The likelihood of each patient having IFD was estimated, and the data were used to construct a receiver operating characteristic (ROC) curve and to calculate the area under the curve (AUC). The cutoff point was determined as the one with the best sensitivity and specificity by calculating the Youden index. A multivariate survival analysis was also performed using the semi-parametric test of the Cox proportional hazards model. The magnitude of the effect was measured by obtaining the HR and its respective 95% CI. The proportionality assumptions were evaluated. The IFD predictor variables from this model were compared with those from the logistic regression model. The RStudio 1.3.1073 (©2009–2020, PBC) program was used for the analyses, a *p*-value < 0.05 was considered significant.

## 3. Results

### 3.1. Characteristics of the Population

Seven hundred seventy-seven episodes of HRFN were evaluated, of which 257 persisted with fever and neutropenia on day 4 of evolution. Of these, 185 were identified as first episodes and included in the study. Of the 185 persistent HRFN episodes assessed, 140 did not progress to IFD, and 45 were classified as IFD (23 proven, 13 probable, and 9 possible). Eleven patients were excluded from the analyses, 9 with possible IFD and 2 with pneumonia due to *Pneumocystis jirovecii*, yielding 34 episodes with proven or probable IFD and 140 episodes without IFD, out of 174 episodes of persistent HRFN. The median age of patients was 7.0 years (IQR 3–13), while the main type of cancer was hematologic malignancy, 85.6% (N = 149 patients), with AML and acute lymphocytic leukemia being the main types of cancer with 35.3% (N = 61 patients) and 29.5% (N = 51 patients), respectively. Antifungal prophylaxis was used in 5 patients (2.9%) during the study period: voriconazole in 3 and fluconazole in 2. Other characteristics of the population are described in Table 1.

Of the 34 episodes with IFD, 18 (52.9%) were filamentous fungi and 16 (47.1%) were yeasts. Among the 21 proven IFD cases, *Candida albicans* (5 cases), *Candida parapsilosis* (4 cases), *Aspergillus* spp. (4 cases), and *Candida tropicalis* (3 cases) predominated, followed by one case each of *Scedosporium* sp., *Candida glabrata*, *Candida lusitaniae*, *Candida dubliniensis*, and *Rhodotorula mucilaginosa* (Table 2). Of the 13 probable cases of IFD, all corresponded to suspected filamentous fungal infection; 11 patients had pulmonary involvement, as demonstrated by computed tomography (CT) scan findings and positive galactomannan (GM) in serum or bronchoalveolar lavage (BAL), and 2 had sinus involvement and compatible biopsy.

### 3.2. Risk Factors

The bivariate analysis showed that patients with IFD were older than patients who did not develop IFD, with a median age of 9.4 years (IQR 5–13) vs. 6 years (IQR 3.1–10.2), respectively (*p* = 0.020). Furthermore, a trend analysis indicated that as the age range increased, so did IFD (*p* = 0.013), with adolescents (47.0%, N = 16) developing more IFD than the rest of the population (Table 1). Clinical and laboratory assessment on admission showed that patients who subsequently developed IFD were admitted to hospital in a more serious condition than those who did not develop a fungal infection, with a higher percentage of patients with hypotension (*p* = 0.033), sepsis (*p* = 0.020), transfer to the Pediatric Intensive Care unit (PICU) (*p* = 0.0001), and CRP ≥ 90 mg/L (*p* = 0.005) (Table 1).

Variables with *p*-value < 0.1 in the bivariate analysis were used to fit a multivariate logistic regression model, defined as the input model. The significant variables in the input model were male sex, adolescent age range compared to infants, CRP ≥ 90 mg/L, transfer to the PICU, sepsis, and hypotension. When the first model (model 1) was adjusted, it was noted that male patients had a higher risk of developing IFD (adjusted OR 4.04; 95% CI 1.58–11.38), while adolescents had a 4.65 times higher risk (95% CI 1.20–21.87) than infants. A CRP ≥ 90 mg/L on admission and transfer to a PICU increases the risk of IFD by 3.13 (95% CI 1.26–8.20) and 10.73 (95% CI 4.26–29.47) times, respectively (Table 3). A second model (model 2) was evaluated by adding the variable type of cancer, considered by the authors as a possible risk factor, with no differences in the ORs of the variables, as shown in Table 3.

**Figure 1:**
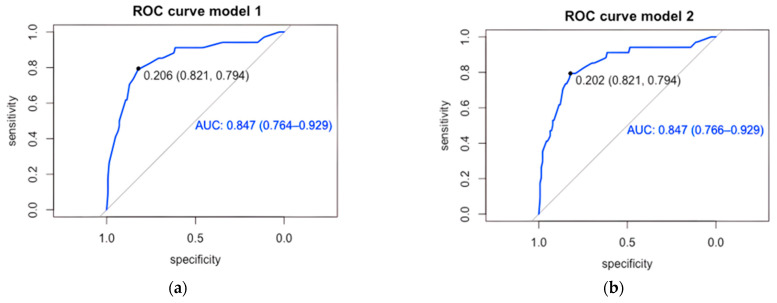
ROC curve of predictive models for invasive fungal disease in children with cancer and episodes of persistent high-risk febrile neutropenia. (**a**) Model 1: Male sex, adolescents, CRP ≥ 90 mg/L and PICU admission, (**b**) Model 2: Male sex, adolescents, CRP ≥ 90 mg/L, PICU admission and type of cancer (hematologic malignance); AUC: area under the curve.

Both models were evaluated for their ability to predict IFD. Model 1, based on the significant variables, discriminated 84.7% (95% CI 76.4–92.9) of patients who developed IFD (Figure 1a), with a sensitivity of 79.4% and specificity of 82.1%, correctly classifying 81.6% of patients at a cutoff value of 0.206. Model 2 (Figure 1b), which included cancer type, showed no differences from model 1 (sensitivity: 79.4%; specificity: 82.1%; correctly classifying 81.1%).

### 3.3. Event-Free Survival

**Figure 2:**
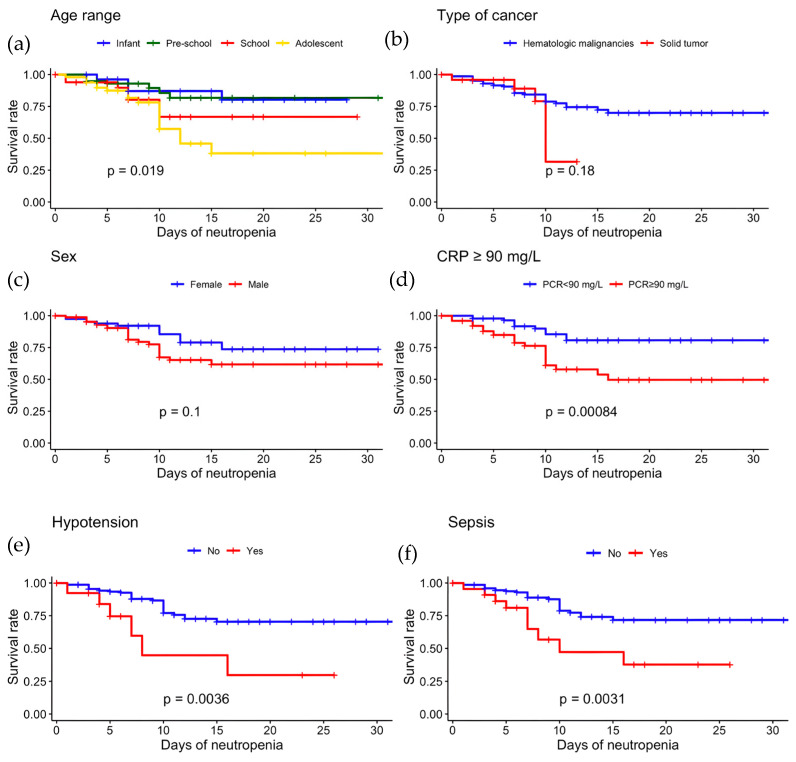
Kaplan–Meier estimator: Survival rates according to: (**a**) age rate; (**b**) type of cancer; (**c**) sex; (**d**) CRP ≥ 90 on admission; (**e**) hypotension; (**f**) sepsis. The *p*-value was obtained using the log-rank test.

The overall event-free survival was 66.7% (95% CI 57.2–77.9%). The highest risk of IFD was observed in the first 7 days of neutropenia. After this period, the risk of developing IFD decreased, and on day 10 of neutropenia, only 66 patients were at risk of developing IFD with an event-free survival of 75% (67–83.9%). Furthermore, by day 10, 85.3% (N = 29) of the IFD cases had accumulated. Kaplan–Meier curves showed that adolescents had a significantly higher risk of developing IFD, with an event-free survival of 38.3% (95% CI 21.3–66.8) compared to other age groups (*p* = 0.015). Other demographic variables, such as cancer type or sex, were not associated with a lower event-free survival, as shown in Figure 2. Patients who developed IFD during their FN episode were admitted in a more serious condition than patients who did not develop IFD. Hypotension, sepsis, and CRP ≥ 90 mg/L were associated with lower event-free survival, results that were statistically significant (Figure 2).

**Figure 3:**
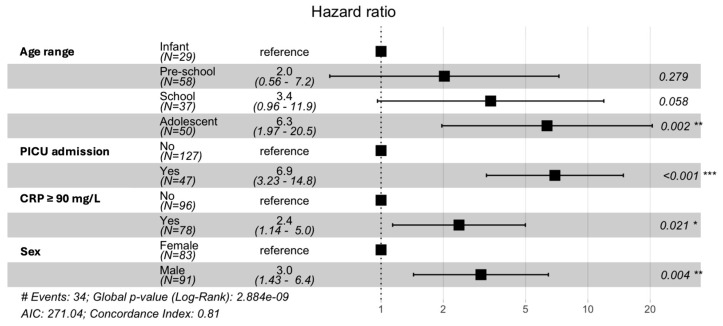
Forest plot and hazard ratio estimation. Results obtained from the analysis of Cox proportional hazards models; Statistically significant variable (*** 0.001; ** 0.01; * 0.05).

A Cox proportional hazard model was adjusted for the significant variables identified in the survival analyses. It was also adjusted for sex, a variable that was not significant in the survival analyses but was considered important for the onset of IFD in previous analyses. As shown in Figure 3, being an adolescent (aged 10 to 18 years) was an independent risk factor and was associated with a 6.3 (95% CI 1.97–20.5) times increased risk of developing IFD compared to younger patients. Being transferred to PICU and having a CRP ≥ 90 on admission were associated with a 6.9 (3.23–14.8) and 2.4 (95% CI 1.14–5.0) times higher risk of IFD, respectively, while male sex was associated with a 3.0 (95% CI 1.43–6.4) times higher risk of developing IFD. These results are consistent with those obtained in the logistic regression analyses.

## 4. Discussion

In our study, we were able to establish that being male, adolescent, and presenting with greater severity at the onset of the FN episode are independent risk factors that can discriminate the development of IFD in pediatric patients with cancer in 84.7% of cases.

Currently, a wide variety of risk factors could serve as predictors of IFD [19,20,21,22,23,24]. This may be mainly due to differences in study designs and populations used to identify factors that predict IFD. A systematic review of 22 studies found considerable heterogeneity across patient populations, including those with HCT and cancer. In addition, the studies assessed risk at different clinical stages and employed diverse methods to measure each factor [3]. Using two multivariate methods, logistic regression and survival analysis, we determined that male sex and age were non-modifiable risk factors for IFD, while greater severity at the onset of FN, as manifested by hypotension, sepsis, PICU admission, and CRP ≥ 90, was independently associated with increased risk of IFD.

**Figure A1:**
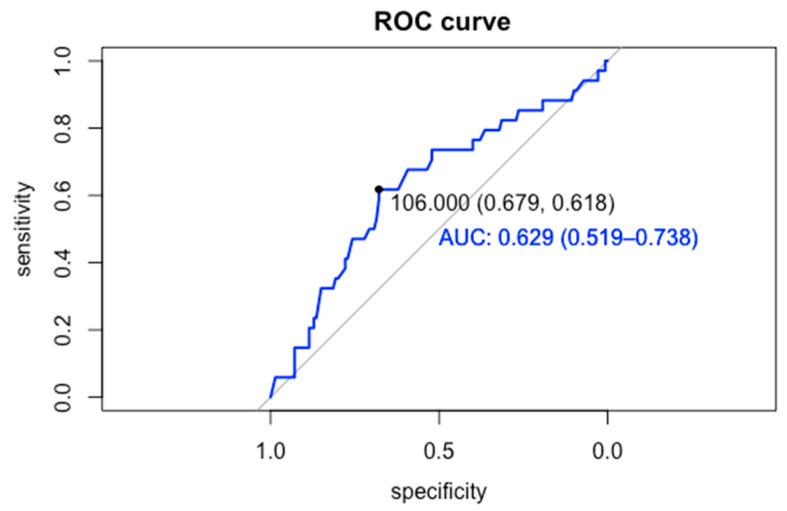
ROC curve of age and age cutoff for invasive fungal infection (106 months = 8.8 years).

Increasing age has been associated with a higher risk of developing IFD in several studies, with 10 years identified as the cutoff [5,6,20,21,24,25]. However, this remains a topic of debate, as it is unclear whether age itself is a risk factor or whether it is associated with such factors as the intensity of chemotherapy, depending on the type of cancer, differences in pharmacokinetics, associated comorbidities, or increased colonization by environmental fungi [3,26]. Our study determined that age was related to the onset of IFD, with a cutoff point for this predictor of 8.8 years (Figure A1), which aligns with the findings of other authors, who found an average age in patients with IFD between 8 and 10 years [6,20,21,24,25]. The group at highest risk of developing IFD in our cohort was adolescents, consistent with the data published by Lehrnbecher et al. [1], where a multivariate analysis showed that the HR for proven/probable IFD increased in patients aged ≥12 years, with a higher proportion of filamentous fungi. These findings are similar to ours, in which the median age of patients with filamentous fungal infections was 11.8 years (IQR 9–13.7), compared with 7.4 years (IQR 2.2–11.5) among children with yeast infections (Table A1). This can be explained by the fact that children, unlike adolescents, develop a stronger adaptive immune response, mainly through T cells (Th17), which promote the elimination of fungi to compensate for an immature innate immune system. This characteristic may make children less likely to develop IFD caused by filamentous fungi than adolescents [12].

Concerning gender, there are currently no data indicating that it could be a risk factor for IFD in pediatric patients. In an observational study of adult patients with myeloid leukemia undergoing induction therapy, Ming-Yu Lien et al. showed that being male was significantly associated with developing IFD [27]. A clinical study conducted on pediatric cancer patients in Chile revealed that 14 of 18 cases of IFD in cancer patients were male, mostly adolescents [4]. Moreover, we noted that the risk of IFD in males increased 4-fold (95% CI: 1.58–11.38) after multivariate analysis. These results may be due to genetic effect-modifying variables that predispose males to a higher risk of IFD. These findings should be reviewed in future studies.

Increased severity on admission has been reviewed in some pediatric studies evaluating predictors of IFD. Monsereenusorn et al. determined that respiratory involvement, the need for oxygen, hypotension, and prolonged hospitalization are associated with IFD [19]. Furthermore, Muayad A. et al. developed a predictive model for IFD, finding that fever and hypotension are significantly associated with its onset [16]. In a recent study, Moraitaki et al. found that ICU hospitalization was associated with an increased risk of IFD in pediatric patients with hematologic cancer [20]. These data, added to the findings of this study, support the hypothesis that severity on admission is a predictive risk factor for IFD in pediatric patients with cancer.

Most studies describing IFD-related risk factors have identified prolonged neutropenia, AML, and ALL relapse as risk factors [3,5,19,21,23,28]. We found no significant differences in these variables because we only worked with the population with persistent HRFN, which occurs mainly in children with hematologic cancer, the population at highest risk for IFD.

In a recent systematic review assessing the main predictors of IFD in pediatric patients, a series of individual risk factors for IFD were identified; however, no prediction rules were found that could be useful for establishing prophylaxis and different antifungal therapy strategies [3]. We established a series of independent risk factors mentioned above and used them to develop a predictive model for IFD in children with cancer and persistent HRFN. This is a simple, intuitive model with readily available clinical parameters, such as age range, sex, CRP ≥ 90, and PICU transfer, to estimate the likelihood of IFD. Our model differentiated 84.7% of patients who developed IFD with good sensitivity and specificity. To date, there are few prediction rules focused on IFD in children with cancer. Muayad A. et al. designed a high-performing IFD risk score (AUC = 0.94), with good sensitivity, specificity, positive predictive value, and negative predictive value [16] using variables as the number of days of fever, the number of days of neutropenia, absolute white blood cell count, hypotension on admission, and the presence of graft-versus-host disease. The variables selected differ from ours mainly in the population studied, since, as previously mentioned, the population in this cohort only includes patients with cancer experiencing persistent HRFN. In contrast, the referred study considers a population of children receiving HCT, who are more immunosuppressed and at greater risk of graft-versus-host disease [16].

This study combined the strengths of a prospective study with a nested retrospective design, offering certain advantages over most retrospective studies. Furthermore, it is a multicenter study, so the likelihood of bias due to factors such as local epidemiology and available diagnostic tools is lower. As a strength, we estimated independent risk factors for IFD using two different methodological approaches, logistic regression and survival analysis, which provide the results obtained with greater robustness and make it possible for us to generate a prediction rule for patients with cancer who develop fever and persistent neutropenia.

An important limitation of this study is that only one episode per patient was used to determine risk factors, which may lead to missing relevant information in the cohort, as intra- and inter-patient variability has been excluded; and that given the limited number of cases, the model’s predictive capacity was assessed within the same cohort. This tool must be validated in a cohort different from the one in which it was created, so new prospective studies are needed to validate and use this prediction rule in the population of children with cancer. Finally, due to the low number of cases, it was not possible to determine distinct independent risk factors for yeast and filamentous fungal infections.

## 5. Conclusions

Identifying risk factors that predict IFD makes it possible to develop tools that can be used as prediction rules. Our study found that patients at the highest risk of developing IFD are adolescents, mainly males, presenting with persistent HRFN who are admitted to the hospital in serious condition, defined in our study as hypotension, sepsis, transfer to PICU, and CRP ≥ 90 mg/L. Creating a predictive model for the risk of IFD in children with cancer can help promote the rational use of antifungals in this population, advancing a personalized approach for this group of patients and aiding in a more rational decision regarding who requires prophylaxis, empirical therapy, or pre-emptive therapy, potentially reducing inappropriate antifungal use, an issue associated with increased toxicity and selection for resistance.

## Figures and Tables

**Table 1:** Demographic and clinical characteristics at admission in 174 children with cancer and episodes of persistent high-risk febrile neutropenia, according to the presence or absence of invasive fungal disease.

Variable (Median; IQR) (N; %)	IFD (N = 34)	No IFD (N = 140)	Total (N = 174)	*p*-Value
Demographic				
Age; years	9.4 (5–13)	6 (3.1–10.2)	7.0 (3–13)	0.0200 *
Male sex	23 (67.7)	68 (48.6)	91 (52.3)	0.0550
Age range				0.0540 **
Infant	4 (11.8)	25 (17.9)	29 (16.7)	Ref
Pre-school	7 (20.6)	51 (36.4)	58 (33.3)	0.8196
School	7 (20.6)	30 (21.4)	37 (21.3)	0.5805
Adolescent	16 (47.0)	34 (24.3)	50 (28.7)	0.0810
Type of cancer				0.5868
Hematologic malignancies	28 (82.4)	121 (86.4)	149 (85.6)	
Solid tumors	6 (17.6)	19 (13.6)	25 (14.4)	
Use of CVC	32 (94.1)	126 (91.3)	158 (91.9)	0.7390
Previous IFD	2 (5.9)	5 (3.6)	7 (4.0)	0.7390
Bacterial co-infection	29 (85.3)	99 (70.7)	128 (73.6)	0.1270
CRP ≥ 90 mg/L	23 (67.6)	55 (39.3)	78 (44.8)	0.0052 *
Hypotension	6 (17.6)	8 (5.7)	21 (12.1)	0.0331 *
Sepsis	9 (26.5)	14 (10)	23 (13.2)	0.0205 *
PICU admission	22(64.7)	25 (17.9)	47(27.0)	0.0001 *

CVC: central venous catheter; IFD: Invasive fungal disease; CRP: C-reactive protein; PICU: pediatric intensive care unit; ref: reference; * Statistically significant variable, ** Trend test (*p* = 0.013).

**Table 2:** Demographic, clinical, imaging, and microbiological characteristics of 34 children with proven or probable Invasive Fungal Disease.

Age (Years)/Gender	Type of Cancer	Clinical Focus and Imaging	Mycological Evidence	Outcome
Proven IFD				
13M	Solid tumor	Renal; CT scan	UC (+) *C. albicans*	Alive
4M	Lymphoma	Renal; CT scan	UC (+) *C. albicans*	Alive
0.8F	AML	Candidemia	BC (+) *C. parapsilosis*	Dead
6M	ALL relapse	Spleen lesions; CT scan	UC (+) *C. glabrata*	Alive
11F	ALL	Renal abscess, CT scan	Renal biopsy; *C. lusitaniae*	Alive
3F	ALL	Candidemia	BC (+) *C. parapsilosis*	Alive
1M	AML	Catheter infection	CVC (+) *C. albicans*	Alive
8M	ALL	Candidemia	BC (+) *C. tropicalis*	Alive
1M	AML	Catheter infection	CVC (+) *C. parapsilosis*	Alive
9F	Solid tumor	Candidemia	BC (+) *C. parapsilosis*	Alive
8M	AML	Hepatosplenic; CT scan	UC (+) *C. albicans*	Alive
13F	Solid tumor	Esophagitis	Esophageal biopsy; *C. dublinensis*	Alive
13M	ALL	Hepatosplenic; CT scan	UC (+) *C. albicans*	Alive
2M	Solid tumor	Candidemia	BC (+) *C. tropicalis*	Alive
0.9M	Solid tumor	Candidemia	BC (+) *C. tropicalis*	Alive
14M	ALL	Fungemia	BC (+) *Rhodotorula mucilaginosa*	Alive
14M	AML	Pneumonia; CT scan	Lung biopsy; *Aspergillus* sp.	Alive
3M	Solid tumor	Pneumonia; CT scan	Serum GM (3.8); (+) PCR *Aspergillus* sp.	Alive
12M	ALL	Pneumonia; CT scan	Lung biopsy; *Aspergillus fumigatus*	Alive
14M	ALL relapse	Pneumonia; CT scan	Sinus biopsy; *Aspergillus* sp.	Alive
13F	ALL	Fungal sinusitis	Sinus biopsy; *Scedosporium* sp., Serum GM (0.7)	Dead
Probable IFD				
8M	AML	Fungal sinusitis	Sinus biopsy; thin septate hyphae	Alive
13M	AML	Fungal sinusitis	Sinus biopsy; septate hyphae with 90-degree bifurcation	Alive
18F	ALL relapse	Pneumonia; CT scan	BAL GM (2.1)	Alive
9M	AML	Pneumonia; CT scan	Serum GM (0.6)	Alive
4M	AML	Pneumonia; CT scan	Serum GM (0.7)	Alive
17M	AML	Pneumonia; CT scan	Serum GM (1.5)	Alive
9M	ALL	Pneumonia; CT scan	Serum GM (0.8)	Alive
9F	ALL	Pneumonia; CT scan	Serum GM (0.6)	Alive
14F	AML	Pneumonia; CT scan	Serum (1.9) and BAL (4.9) GM	Alive
6M	ALL	Pneumonia; CT scan	BAL GM (4.1)	Alive
11M	AML	Pneumonia; CT scan	Serum GM (1.1)	Alive
12F	ALL	Pneumonia; CT scan	Serum (2.8) and BAL (6.5) GM (+)	Alive
11F	ALL	Pneumonia; CT scan	Serum GM (0.8)	Dead

AML: acute myeloid leukemia; ALL: acute lymphoblastic leukemia; M: male; F: female; CVC: central venous catheter; UC: urine culture; BC: blood culture; GM: galactomannan antigen (serum > 0.5; BAL ≥ 1); BAL: bronchoalveolar lavage; CT: computed tomography.

**Table 3:** Risk factors for invasive fungal diseases in children with cancer and episodes of persistent high-risk febrile neutropenia obtained by multivariate logistic regression analysis.

Variable	Input Model		Model 1		Model 2	
	Crude OR	95% CI	Adjusted OR	95% CI	Adjusted OR	95% CI
Male sex	2.21	1.02–5.04	4.04	1.58–11.38	4.04	1.58–11.36
Age range (ref Infant)						
Adolescent	2.94	0.94–11.23	4.65	1.20–21.87	4.74	1.21–22.61
CRP ≥ 90 mg/L	3.23	1.49–7.39	3.13	1.26–8.20	3.05	1.20–8.15
Hypotension	3.54	1.09–10.98				
Sepsis	3.24	1.23–8.25				
PICU admission	8.43	3.76–19.81	10.73	4.26–29.47	10.65	4.35–29.28
Type of cancer (ref hematologic malignancy)	1.36	0.46–3.57			1.18	0.32–3.97

CRP: C-reactive protein; PICU: pediatric intensive care unit; ref: reference.

## Data Availability

No new data were created or analyzed in this study. Data sharing is not applicable to this article.

## Abbreviations

The following abbreviations are used in this manuscript:

IFD	Invasive fungal disease
CRP	C-reactive protein
RF	Risk factor
HRFN	high-risk febrile neutropenia
PICU	Pediatric intensive care unit
AML	Acute myeloid leukemia
HCT	Hematopoietic cell transplant
PINDA	National child program of antineoplastic drugs
IQR	Interquartile range
CI	confidence intervals
EFS	Event-free survival
HR	Hazard ratio
ROC	Receiver operating characteristic
CVC	Central venous catheter
GM	Galactomannan
CT	Computed tomography
BAL	Bronchoalveolar lavage
FB	Fungus ball
UC	Urine culture
BC	Blood culture

## Appendix A

**Table A1:** Comparison between filamentous fungal infections and yeast infections in children with cancer and episodes of persistent high-risk febrile neutropenia.

Variables Median (IRQ), N (%)	Mold Infections (N = 18)	Yeast Infections (N = 16)	*p*-Value
Age (Years)	11.8 (9.0–13.7)	7.4 (2.2–11.5)	0.0198
Age Range			0.0929
Infant	0	4 (25.0)	
Pre-school	3 (16.7)	4 (25.0)	
Elementary School	4 (22.2)	2 (18.8)	
Adolescent	11 (61.1))	5 (31.2)	
Male sex	12 (66.7)	11 (68.8)	1
Hematologic Malignancies	17 (94.4)	11(68.7)	0.0782
Solid tumors	1 (5.6)	5(31.3)	
CRP ≥ 90 mg/L	11 (61.1)	12 (75.0)	0.4768
Hypotension	4 (22.2)	1(12.5)	0.6602
Sepsis	6 (33.3)	6 (37.5)	1
PICU admission	12 (66.7)	10 (62.5)	1
